# One-step hydrothermal synthesis of a ternary heterojunction g-C_3_N_4_/Bi_2_S_3_/In_2_S_3_ photocatalyst and its enhanced photocatalytic performance†

**DOI:** 10.1039/d1ra00729g

**Published:** 2021-03-05

**Authors:** Teng Zhao, Xiaofeng Zhu, Yufan Huang, Zijun Wang

**Affiliations:** School of Chemistry and Chemical Engineering, Shihezi University Beisi Road Shihezi Xinjiang 832003 PR China wzj_tea@shzu.edu.cn +86 15699322089; Key Laboratory for Green Processing of Chemical Engineering of Xinjiang Bingtuan, Key Laboratory of Materials-Oriented Chemical Engineering of Xinjiang Uygur Autonomous Region, Engineering Research Center of Materials-Oriented Chemical Engineering of Xinjiang Bingtuan Shihezi Xinjiang 832003 PR China

## Abstract

In recent years, photoelectrocatalysis has been one of the hotspots of research. Graphite-like carbon nitride (g-C_3_N_4_) is one of the few non-metal semiconductors known and has good potential in the field of photocatalysis due to its simple preparation method and visible light effects. In this study, a method for compounding two semiconductor materials, In_2_S_3_ and Bi_2_S_3_, on the surface of g-C_3_N_4_*via* a one-step hydrothermal method is reported, and it was found that this resulting material showed remarkable properties. The advantages of this method are as follows: (1) the formation of a heterojunction, which accelerates the separation efficiency of photogenerated carriers; (2) a large number of holes and defects on the surface of g-C_3_N_4_ are conducive to the nucleation, crystallisation and growth of In_2_S_3_ and Bi_2_S_3_. Compared with its counterpart catalysts, the CN/In_2_S_3_/Bi_2_S_3_ composite catalyst has significantly improved performance. Due to its high degree of crystallinity, the adsorption capacity of the catalyst itself is also significantly improved. In addition, the stability of the composite material maintains 90.9% after four cycles of use, and the structure is not damaged. In summary, CN/Bi_2_S_3_/In_2_S_3_ composite materials are believed to have broad application potential in the treatment of dye wastewater.

## Abstract 2

The proposed photocatalytic mechanism for the degradation of RhB on the surface of the 0.05CN/Bi_2_S_3_/In_2_S_3_ composite.

## Graphical abstract

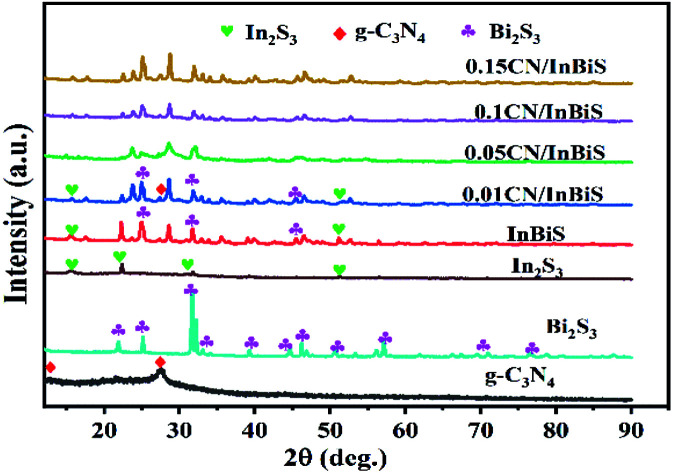

## 1 Introduction

With the development of science and technology, the application of photoelectrocatalytic technology^1–5^ is of great benefit to the water pollution caused by the of the global industrialization process.^6–9^ As a result, different countries and regions worldwide have made great efforts to protect the environment on which people depend.^10–12^ Fortunately, at the same time, there have been continuous developments in science and technology toward alleviating pollution in water. Common methods that have been developed for dealing with organic pollution in water can be divided into three categories: physical, chemical and biological methods. Adsorption is the main physical method used,^13^ which mostly involves the use of activated carbon and diatomaceous earth as adsorbents. Other adsorbents have been also reported recently for wastewater treatment, *e.g.*, sepiolite mineral nanofibers.^14^ However, the costs associated with this method are high and complete adsorption of the pollutants from water cannot be achieved. Oxidation reactions are the main chemical methods used in the remediation of water pollutants.^15,16^ Therefore, it is urgent to develop an environmentally friendly method to solve the current water pollution issues. As photocatalytic technology has the advantages of being green, highly efficient and causing no secondary pollution, it is one of the most promising methods for degrading pollutants in water that could be applied as a future strategy.^17–19^

At present, the reports on photocatalysts in the literature are mainly based on semiconductor materials, including titanium dioxide (TiO_2_),^20^ molybdenum sulfide (MoS_2_),^21^ Bi_2_S_3_,^22^ zinc oxide (ZnO),^23^ graphitic carbon nitride (g-C_3_N_4_).^24^ The unique forbidden band structures of semiconductors lead to the generation of electrons and holes (e^−^ and h^+^) under the excitation of light of a certain wavelength. These e^−^ and h^+^ then react with water molecules and oxygen in water to form strong oxidising substances, which oxidise and decompose the pollutants present in water into environmentally friendly molecules. Among these semiconductors, in 2009 g-C_3_N_4_ was first used by Wang *et al.* in the photocatalytic production of hydrogen. Due to the excellent photoelectric properties of g-C_3_N_4_, it has attracted the attention of many researchers in the field of photocatalysis.^19,24^ In addition to photocatalytic application,^25^ graphitic carbon nitride based materials are also promising for biomedical applications.^26^ However, some studies have shown that the rapid recombination of e^−^ and h^+^ severely limits the photocatalytic performance of g-C_3_N_4_.^27^ To solve and overcome these difficulties, many researchers have made tremendous efforts,^28,29^ such as using different strategies to improve the catalytic performance of g-C_3_N_4_. For example, a higher number of active sites have been introduced in g-C_3_N_4_ by increasing its specific surface area,^30^ however, the coverage of dyes and other small molecules on the catalyst surface and in the pores makes the catalyst less stable. The latest research shows that a strategy of constructing heterojunction binary and multi-component catalysts not only accelerates the separation of e^−^ and h^+^ but also expands the range of light response of these materials has attracted great attention.^31–33^ It has been reported in the literature that the construction of heterostructured catalysts leads to systems that have greatly improved photocatalytic performance.^34–37^ C. Auttaphon *et al.*^38^ synthesised a Z-scheme Bi_2_S_3_/ZnIn_2_S_4_ heterostructure for methylene blue degradation, which showed higher photocatalytic activity than the uncombined catalysts alone. Geioushy *et al.* used a one-pot method to prepare BiPO_4_/Bi_2_S_3_ for the reduction of harmful Cr(vi) driven by visible light.^39^ ABi_2_S_3_/Bi_2_W_2_O_9_ composite has also previously been prepared that shows 80% degradation of phenol after 120 min of irradiation, a performance that is substantially higher than those of pure Bi_2_S_3_ and Bi_2_W_2_O_9_ under visible-light illumination.^40^ An *et al.* synthesised a new Z-scheme In_2_S_3_/graphene heterojunction with a core–shell structure, which showed a methyl orange degradation rate of almost five times higher than that of pure In_2_S_3_ (ref. 41) and reduced Cr(vi) under visible light. For degradation of rhodamine B (RhB), these photocatalysts have been reported recently.^42,43^ In summary, from the perspective of development, photocatalytic technology has great application prospects in the degradation of pollution and environmental protection.^44,45^

In this study, to verify the heterostructure photocatalyst theory, the ternary heterostructured photocatalyst g-C_3_N_4_/Bi_2_S_3_/In_2_S_3_ was prepared *via* a one-step hydrothermal method. The modification of microscale porous spherical Bi_2_S_3_ and dendritic In_2_S_3_ on the surface of porous g-C_3_N_4_ with a large specific surface area significantly improves its optical, physical and photoelectric conversion properties. Compared with the performances of the g-C_3_N_4_, Bi_2_S_3_ and In_2_S_3_ catalysts alone, and Bi_2_S_3_/In_2_S_3_ binary heterojunction catalysts, the obtained g-C_3_N_4_/Bi_2_S_3_/In_2_S_3_ ternary heterostructured photocatalyst shows significantly improved degradation of rhodamine B (RhB). In addition, by carrying out powder X-ray diffraction (XRD), photoluminescence (PL), X-ray photoelectron spectroscopy (XPS) and other characterisation methods, the composition of the catalyst in terms of its elemental content, its chemical valence state, morphology, and charge carrier transfer behaviour were carefully analysed, and the possible catalytic mechanism of the catalyst was explored and is discussed in detail. This work may provide a feasible practical example of a way of improving the photocatalytic performance of g-C_3_N_4_ through its combination with Bi_2_S_3_ and In_2_S_3_ in a heterogeneous composition as a reference for subsequent research.

## 2 Experimental

### 2.1 Materials

Thiourea, absolute ethanol, RhB, urea, bismuth nitrate pentahydrate, indium nitrate, and other chemicals used in the experiments were of analytical purity (99%) and used without further purification. Deionised water was used throughout all of the experimental work in this study. All medicines come from Titan.

### 2.2 Sample preparation

#### 2.2.1 Preparation of the g-C_3_N_4_ photocatalyst

An appropriate amount of urea was added to a crucible and closed the lid, and heated in a muffle furnace at a heating rate of 2.5 °C min^−1^ to 550 °C, and held at this temperature for 4 h to obtain a yellow solid of g-C_3_N_4_, which was ground into a powder for subsequent use.

#### 2.2.2 Pretreatment scheme of g-C_3_N_4_

An appropriate amount of g-C_3_N_4_ powder was added to a 50 mL beaker, into which 20 mL of DMF (*N*,*N*-dimethylformamide) was poured and the reaction mixture was stirred at room temperature for 12 h to prepare a solution referred to as solution A.

#### 2.2.3 Preparation of g-C_3_N_4_/Bi_2_S_3_/In_2_S_3_

70 mL of HON_3_ solution with a concentration of 1 mol L^−1^ was added to a 100 mL beaker and stirred for ten min before adding 0.1 mmol of Bi (NO_3_) 5H_2_O, and stirring the resulting reaction mixture for 30 min. Then, to this mixture 0.1 mmol of In (NO_3_) *x*H_2_O was added and the mixture was stirred for 30 min. After this time 0.2 g of cetrimonium bromide (CTAB) was added and the reaction mixture was ultrasonicated for 30 min, to produce a solution referred to as solution B. The above solution A was slowly added dropwise to solution B to mix them together uniformly, then 0.6 g of thiourea was added and the reaction mixture was stirred for 30 min, before being poured into a 100 mL polytetrafluoroethylene liner and heated to 140 °C under hydrothermal reaction for 12 h. The resulting liquid was washed three times with ethanol and deionised water, and then dried in an oven overnight at 60 °C. g-C_3_N_4_/Bi_2_S_3_/In_2_S_3_ catalysts with different mass fractions of g-C_3_N_4_ were prepared, and the catalysts g-C_3_N_4_ and Bi_2_S_3_/In_2_S_3_ were prepared for comparison.

### 2.3 Characterisation of the photocatalysts

XRD patterns were recorded using a Bruker D8 Advance powder X-ray diffractometer equipped with a Cu-Kα radiation source. A Nicolet iS10 Fourier-transform infrared (FTIR) spectrometer was employed to record FTIR spectra of the samples in the wavenumber range of 500–4000 cm^−1^. XPS measurements of the prepared samples were recorded using a PHI 5000 Versa Probe spectrometer, in which the binding energy positions were calibrated against the C–C bond peak that has a binding energy of 284.8 eV. The morphology characteristics of the samples were investigated using transmission electron microscopy (TEM, Tecnai G F20, Hitachi, HT7700) and scanning electron microscopy (SEM, Hitachi S-4800). UV-visible diffuse reflection spectroscopy (DRS) measurements were carried out using a UV-visible spectrophotometer (Shimadzu UV-3600) across a wavelength range of 200–800 nm, employing BaSO_4_ as a benchmark. Fluorescence emission and time-resolved fluorescence spectra were measured using a fluorescence spectrometer (Hitachi F-7000) over a wavelength range of 340–800 nm at an excitation wavelength of 320 nm, employing a 300 W xenon lamp (CEL-HXF300) as a light source.

### 2.4 Photocatalytic activity

To determine the photocatalytic activity of the synthesised photocatalyst, the catalytic degradation of RhB was evaluated under simulated sunlight using a 500 W xenon lamp as a light source in a photoreactor (CEL-HXF300, Beijing, China). In a typical experiment, before being exposed to light the reactants and catalyst were placed in a quartz tube and magnetically stirred for 30 min in the dark to achieve adsorption–desorption equilibrium. At a given time interval after beginning the visible light irradiation, samples were taken from the reaction suspension (each with a volume of 5 mL), centrifuged at 8000 rpm for 10 minutes, and then the supernatant was removed. Then, the concentration of RhB in the sample solutions was analysed using a (UV-31/32/3300) UV-visible spectrometer (MAPADA) at its maximum absorption wavelength. To ensure the repeatability of the results, each run was repeated to obtain an average for each set of conditions. A blank test was also carried out by irradiating the reactant solution in the absence of the catalyst to evaluate its photo-induced self-sensitised photodegradation.
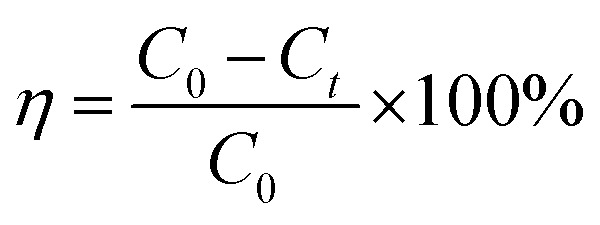
where *η* is the photocatalytic efficiency; *C*_0_ is the concentration of the reactant before illumination and *C*_*t*_ is the concentration of the reactant after illumination for *t* hour.

## 3 Results and discussion

### 3.1 Morphology and structure

#### 3.1.1 XRD analysis

**Fig. 1:**
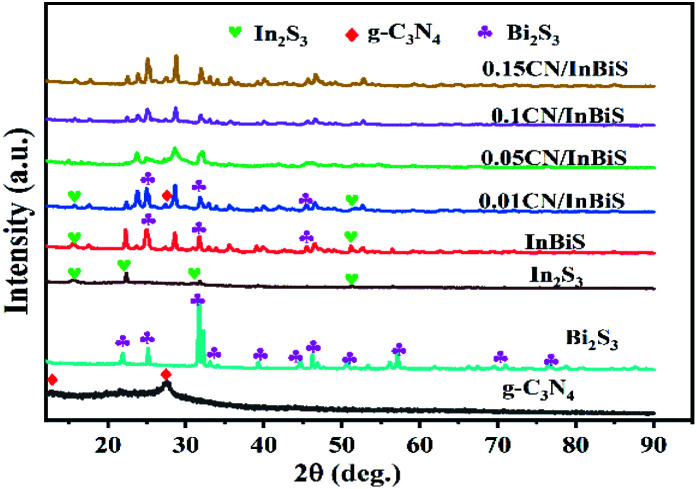
The typical XRD patterns of CN, Bi_2_S_3_/In_2_S_3_ and CN/Bi_2_S_3_/In_2_S_3_.

As shown in Fig. 1, XRD was conducted to identify the crystallographic and phase structures of Bi_2_S_3_, CN, In_2_S_3_, Bi_2_S_3_/In_2_S_3_ and CN-*x*Bi_2_S_3_/In_2_S_3_. The XRD pattern of pure CN shows two distinct diffraction peaks at 2*θ* = 12.43° and 27.54°, which can be indexed to the (002) and (100) diffraction planes, respectively, of g-C_3_N_4_ (JCPDS (87-1526)), attributed to its typical stacked aromatic rings and interlayer π–π stacking.^46^Fig. 1 shows distinct diffraction peaks at 2*θ* = 11.14°, 15.80°, 15.90°, 17.58°, 22.39°, 23.72°, 24.92°, 25.20°, 28.60°, 31.65°, 31.79°, 32.93°, corresponding to the (110), (020), (200), (120), (220), (101), (130), (310), (211), (040), (221), (301) diffraction planes respectively, of Bi_2_S_3_ (JCPDS No. 17-0320).^22^ The profile of In_2_S_3_ exhibits the characteristic diffraction peaks of β-In_2_S_3_ (JPCDS No. 65-0459).^47^ However, the XRD pattern of In_2_S_3_/Bi_2_S_3_ only shows the diffraction peaks of Bi_2_S_3_, showing that it contains little In_2_S_3_ content. In addition, one new weak characteristic peaks at 27.54° is present in the patterns of the CN/Bi_2_S_3_/In_2_S_3_ composites, which can be assigned to the (0 0 2) peak of g-C_3_N_4_. With an increase in the amount of g-C_3_N_4_ present in the catalyst samples, the diffraction peak of Bi_2_S_3_/In_2_S_3_ at 2*θ* = 24.92° went from being of high intensity to low intensity, reflecting the influence that it has on the phase crystallinity of Bi_2_S_3_/In_2_S_3_.

#### 3.1.2 SEM/TEM analysis

**Fig. 2:**
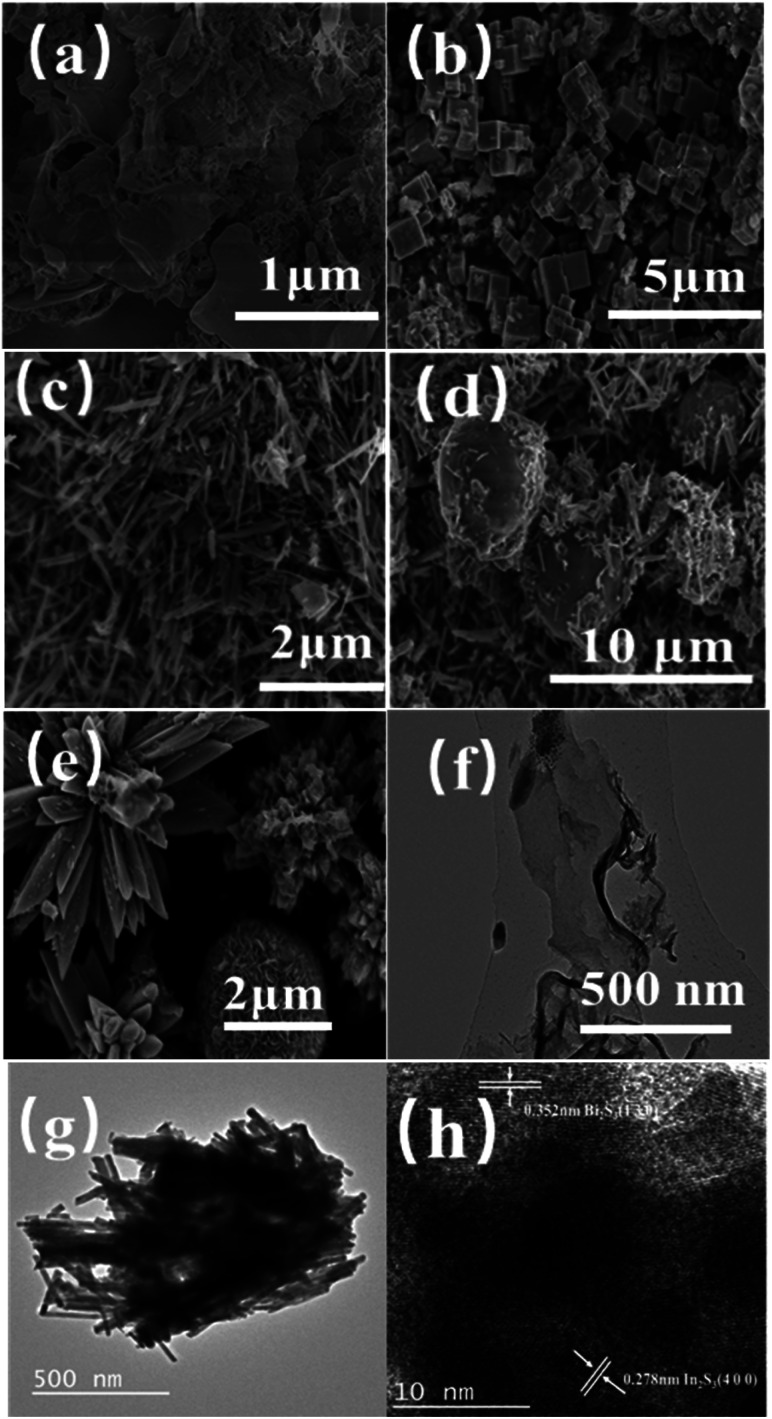
SEM images of (a) g-C_3_N_4_; (b)In_2_S_3_; (c) Bi_2_S_3_; (d) Bi_2_S_3_/In_2_S_3_; (e) 0.05CN/Bi_2_S_3_/In_2_S_3_; TEM images of (f) g-C_3_N_4_; (g) Bi_2_S_3_/In_2_S_3_ and HRTEM images of (h) 0.05CN/Bi_2_S_3_/In_2_S_3_.

In Fig. 2a the layered porous structure of g-C_3_N_4_ can be seen in CN-*x*Bi_2_S_3_/In_2_S_3_, which is beneficial for the adsorption of dyes by the catalyst. Fig. 2b shows an image of the structure of In_2_S_3_, which is made up of a large number of hexagons with side lengths ranging from 0.3–1 μm. Fig. 2c shows an image of Bi_2_S_3_, which has a rod-shaped structure, where the rods have diameters in the range of 50–100 nm and a length in the range of 0.5–2.0 μm in size.

Compared with pure In_2_S_3_ and Bi_2_S_3_, the morphology of the composite of both materials in Fig. 2d shows a spherical structure of In_2_S_3_ surrounded by rod-shaped Bi_2_S_3_. This structure is more conducive to the transfer of photogenerated e^−^ and h^+^ than the single catalysts alone. Fig. 2e shows an SEM image of 0.05CN/Bi_2_S_3_/In_2_S_3_, which shows the combination of flower-like In_2_S_3_ and a spherical Bi_2_S_3_ structure. Porous layered g-C_3_N_4_ has a large specific surface area and pores, so it provides more defects with which to facilitate the crystallisation, nucleation and growth of In_2_S_3_ and Bi_2_S_3_. In addition, the structures of In_2_S_3_ and Bi_2_S_3_ are not damaged. To analyse its structure in more depth, TEM and high-resolution TEM (HRTEM) were used to characterise 0.05CN/Bi_2_S_3_/In_2_S_3_. Fig. 2f shows a TEM image of g-C_3_N_4_, from which it can be seen that the layered g-C_3_N_4_ of the film structure is consistent with the results presented in the literature. Fig. 2g shows a TEM image of Bi_2_S_3_/In_2_S_3_, which is consistent with the image shown in Fig. 2d. Fig. 2h shows a HRTEM image of 0.05CN, in which it can be clearly seen that there are two lattice fringes with different widths of 0.352 and 0.278 nm, corresponding to the (1 3 0) crystal plane of Bi_2_S_3_ and the (4 0 0) crystal plane of In_2_S_3_. This result is consistent with the PXRD results.

#### 3.1.3 FTIR analysis

**Fig. 3:**
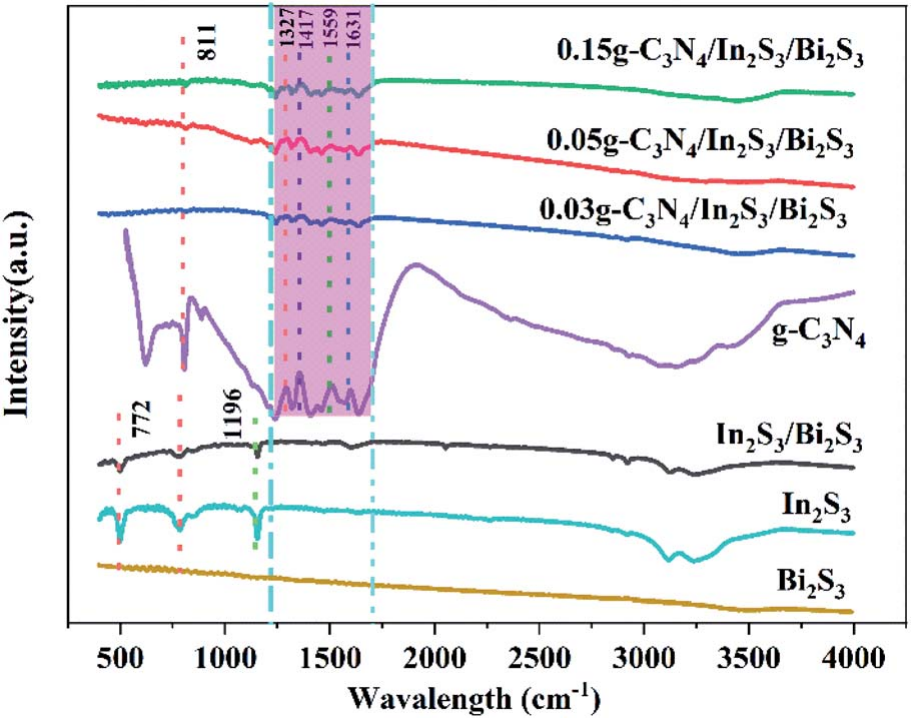
The FTIR spectra of CN, Bi_2_S_3_/In_2_S_3_ and CN-*x*Bi_2_S_3_/In_2_S_3_.

To further demonstrate the surface properties and functional groups present in the samples, FTIR spectra of In_2_S_3_, Bi_2_S_3_, g-C_3_N_4_ and CN-*x*Bi_2_S_3_/In_2_S_3_ were recorded and the results are presented in Fig. 3. In the FTIR spectrum of CN-*x*Bi_2_S_3_/In_2_S_3_ there is no obvious peak that is characteristic of Bi_2_S_3_.^48^ The peaks at 1327, 1417, 1559, and 1631 cm^−1^ in the same spectrum can be assigned as the characteristic absorption bands of g-C_3_N_4_, attributed to the vibration of the C_6_N_7_ skeleton and the typical stretching mode of the CN heterocycles.^49^

The peak at around 809 cm^−1^ can be assigned to the breathing mode of the triazine units. In addition, the peaks at 772 and 1196 cm^−1^ are typical of In_2_S_3_ and the characteristic peak of Bi_2_S_3_/In_2_S_3_ is roughly consistent with In_2_S_3_.^50,51^ The characteristic absorption peak of g-C_3_N_4_/In_2_S_3_/Bi_2_S_3_ is consistent with that of g-C_3_N_4_, while the peak related to Bi_2_S_3_/In_2_S_3_ gradually decreases in intensity, which may be a result of the g-C_3_N_4_ peak being too intense.

#### 3.1.4 XPS analysis

**Fig. 4:**
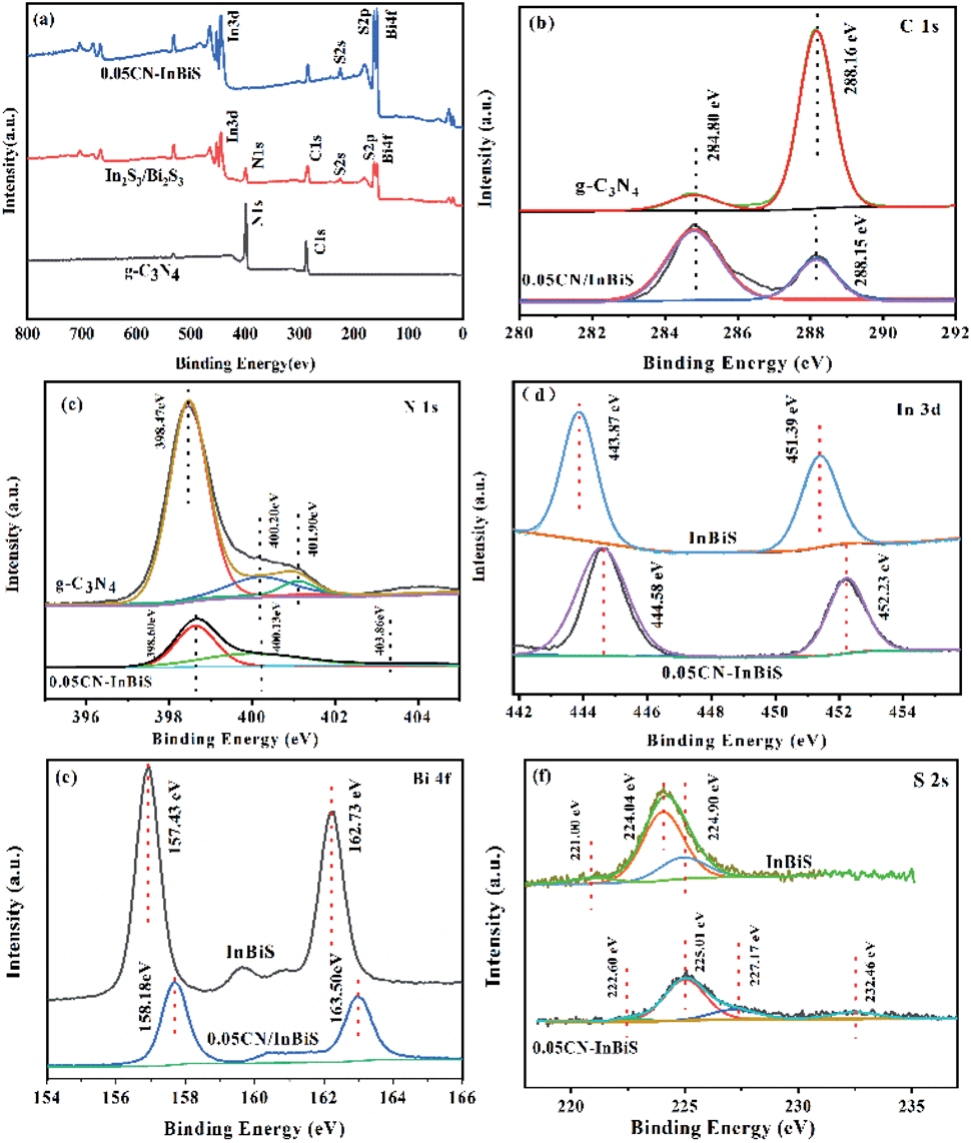
XPS spectra of the CN, In_2_S_3_/Bi_2_S_3_, and 0.05CN/In_2_S_3_/Bi_2_S_3_; (a) survey, (b) C 1s, (c) N 1s, (d) In 3d, (e) Bi 4f, and (f) S 2s spectra.

To study the surface chemical states and chemical compositions of the CN/Bi_2_S_3_/In_2_S_3_ heterojunction samples, Fig. 4 shows XPS measurements of the samples, in which it can be seen that the survey spectrum of CN–In_2_S_3_/Bi_2_S_3_ exhibits the typical C 1s, S 2p, S 2s, N 1s, In 3d, and Bi 4f peaks expected for CN–Bi_2_S_3_/In_2_S_3_, Bi_2_S_3_/In_2_S_3_ and CN. The survey spectrum indicates that C and N exist on the surface of g C_3_N_4_, and Bi, In, and S on the surface of In_2_S_3_/Bi_2_S_3_. Besides this, as shown in Fig. 4a, Bi, In and S were found to be present on the surface of the g-C_3_N_4_ nanosheets, since the peaks at around 443.61, 157.43, and 225.01 eV can be assigned to the In 3d, Bi 4f and S 2s energy levels, respectively. The high-resolution C 1s, N 1s, In 3d, Bi 4f and S 2s XPS spectra of 0.05CN/Bi_2_S_3_/In_2_S_3_ are presented in Fig. 4b–f. Moreover, the binding energy peak of the N–C=N groups in the 0.05CN–Bi_2_S_3_/In_2_S_3_ heterostructure shifts lowers by 0.02 eV to 288.14 eV, which indicates that there are weak interactions between the Bi_2_S_3_/In_2_S_3_ and g-C_3_N_4_ nanosheets as a result of surface modification effects.^52^ The N 1s Gaussian curve in Fig. 4b can be deconvolved into three peaks that have binding energies of 398.47, 400.20, and 401.90 eV, corresponding to sp^2^-hybridised nitrogen (C–N=C), the N–(C)3 groups of the skeleton, and the surface uncondensed bridging N atoms with C–N–H functional groups attached.^53,54^ Compared to g-C_3_N_4_, the peak positions in the N 1s spectrum of 0.05CN/Bi_2_S_3_/In_2_S_3_ are shifted to higher binding energies. In the In 3d spectrum (Fig. 4d), the In 3d_3/2_ and In 3d_5/2_ peaks of 0.05CN/Bi_2_S_3_/In_2_S_3_ are located at 452.23 and 444.58 eV, which indicates a slight shift to higher binding energies compared to pristine In_2_S_3_ (451.39 and 443.87 eV). As shown in Fig. 4e, the Bi 4f XPS spectrum of Bi_2_S_3_ displays clearly two peaks (162.73 and 157.43 eV), which can be attributed to the 4f_5/2_ and 4f_7/2_ of Bi^3+^. Moreover, compared to Bi_2_S_3_, the peak positions (163.50 and 158.18 eV) of Bi 4f in 0.05CN/Bi_2_S_3_/In_2_S_3_ are shifted to higher binding energies. Since the peak of Bi 4f covers the characteristic peak of S 2p, S 2s is added to illustrate the existence of S^2−^. As shown in Fig. 4f, the S 2s XPS spectrum of Bi_2_S_3_/In_2_S_3_ shows three peaks at 221.0, 224.0, and 224.9 eV. Compared with Bi_2_S_3_/In_2_S_3_, 0.05CN/Bi_2_S_3_/In_2_S_3_ shows peaks that are slightly shifted to higher binding energies (222.6, 225.01, and 227.17 eV). These results suggest the successful heterogeneous growth of In_2_S_3_ and Bi_2_S_3_ on the g-C_3_N_4_ nanosheets.

#### 3.1.5 UV-vis analysis

**Fig. 5:**
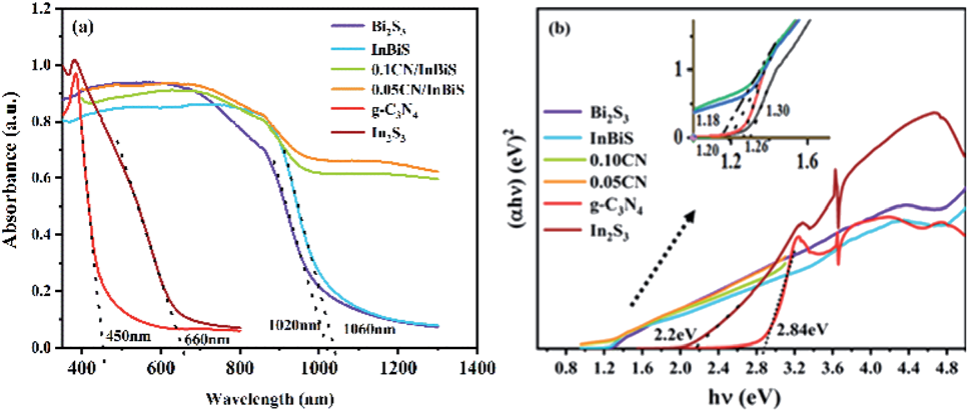
(a) UV-vis DRS of g-C_3_N_4_, In_2_S_3_, Bi_2_S_3_, Bi_2_S_3_/In_2_S_3_, 0.01CN/Bi_2_S_3_/In_2_S_3_, and 0.05CN/Bi_2_S_3_/In_2_S_3_ and (b) plot of (*αhv*)^*n*/2^*versus hv*.

To investigate the modification effect that the g-C_3_N_4_ nanosheets have on the Bi_2_S_3_/In_2_S_3_ heterostructure, the optical absorption properties of the as-prepared samples were analysed using UV-vis spectroscopy, the results of which are shown in Fig. 5a. The g-C_3_N_4_ shows good light absorption at a wavelength of less than 450 nm. The light absorption of In_2_S_3_ and Bi_2_S_3_ seem much higher than that of g-C_3_N_4_, at a wavelength of 1000 nm. Compared with Bi_2_S_3_/In_2_S_3_, the absorption edge of the g-C_3_N_4_/Bi_2_S_3_/In_2_S_3_ heterostructure exhibits a distinct red shift, which may originate from the modification effect of Bi_2_S_3_/In_2_S_3_ on the surface of the g-C_3_N_4_ nanosheets. This signifies that a strong interaction is formed between g-C_3_N_4_ and Bi_2_S_3_/In_2_S_3_, which is beneficial to improving the separation efficiency of charge carriers and the stability of the heterostructure. In addition, the band gap energies (*E*_g_) of the samples were calculated using the Tauc equation:(*αhν*)^2^ = *A*(*hν* − *E*_g_)^*n*^where *α*, *hν*, *A* and *E*_g_ are the absorption coefficient, photoenergy, proportionality constant and band gap, respectively. As shown in Fig. 5b, the band gaps of g-C_3_N_4_, In_2_S_3_ and Bi_2_S_3_ are 2.84, 2.2, and 1.30 eV, respectively. In addition, the band gap of Bi_2_S_3_/In_2_S_3_ is estimated to be 1.26 eV, and the band gaps of 0.01CN/Bi_2_S_3_/In_2_S_3_ and 0.05CN/Bi_2_S_3_/In_2_S_3_ are 1.20 and 1.18 eV, respectively. Compared with Bi_2_S_3_/In_2_S_3_, the band gap of 0.05CN/Bi_2_S_3_/In_2_S_3_ is narrower, which indicates that the heterostructure of 0.05CN/Bi_2_S_3_/In_2_S_3_ is more easily excited by visible light and is beneficial for improving photocatalytic performance due to the modification of large holes and defects on the surface of the g-C_3_N_4_ nanosheets.

#### 3.1.6 PL spectra and photocurrent analysis

**Fig. 6:**
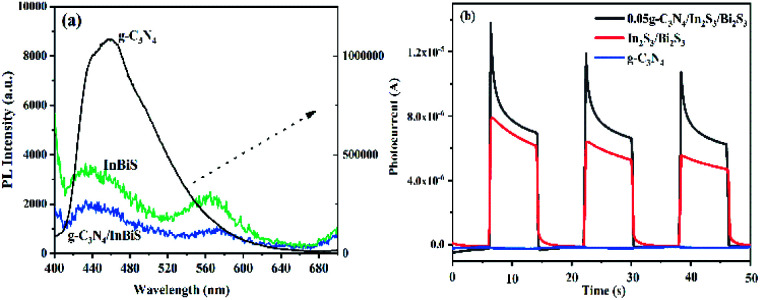
PL spectra (a) and photocurrent (b) of the g-C_3_N_4_, Bi_2_S_3_/In_2_S_3_ and 0.05 g-C_3_N_4_/Bi_2_S_3_/In_2_S_3_ composites.

To further understand the reasons for the improvement in the photocatalytic activity of the composite photocatalyst, the PL spectra were measured to investigate the separation efficiency of photo-induced charge carriers.^55^ The PL spectra of g-C_3_N_4_, Bi_2_S_3_/In_2_S_3_ and 0.05 g-C_3_N_4_/Bi_2_S_3_/In_2_S_3_ are shown in Fig. 6a. The PL emission stems from the radiative recombination between the excited e^−^ and h^+^ and the weaker the PL peak intensity is, the slower the recombination rate of the excited e^−^ and h^+^. Comparing the PL emission spectra of the three materials, the 0.05 g-C_3_N_4_/Bi_2_S_3_/In_2_S_3_ heterostructure shows low fluorescence intensity relative to that shown by g-C_3_N_4_ and Bi_2_S_3_/In_2_S_3_. These results indicate that the efficient interfacial charge transfer between Bi_2_S_3_/In_2_S_3_ and g-C_3_N_4_ hinders the recombination of e^−^ and h^+^ pairs. In addition, Fig. 6b is the photocurrent curve of the catalysts. We can clearly see that the photocurrent value of the composite material is the highest, and the conclusion corresponds to Fig. 6a.

### 3.2 Photocatalytic activity

**Fig. 7:**
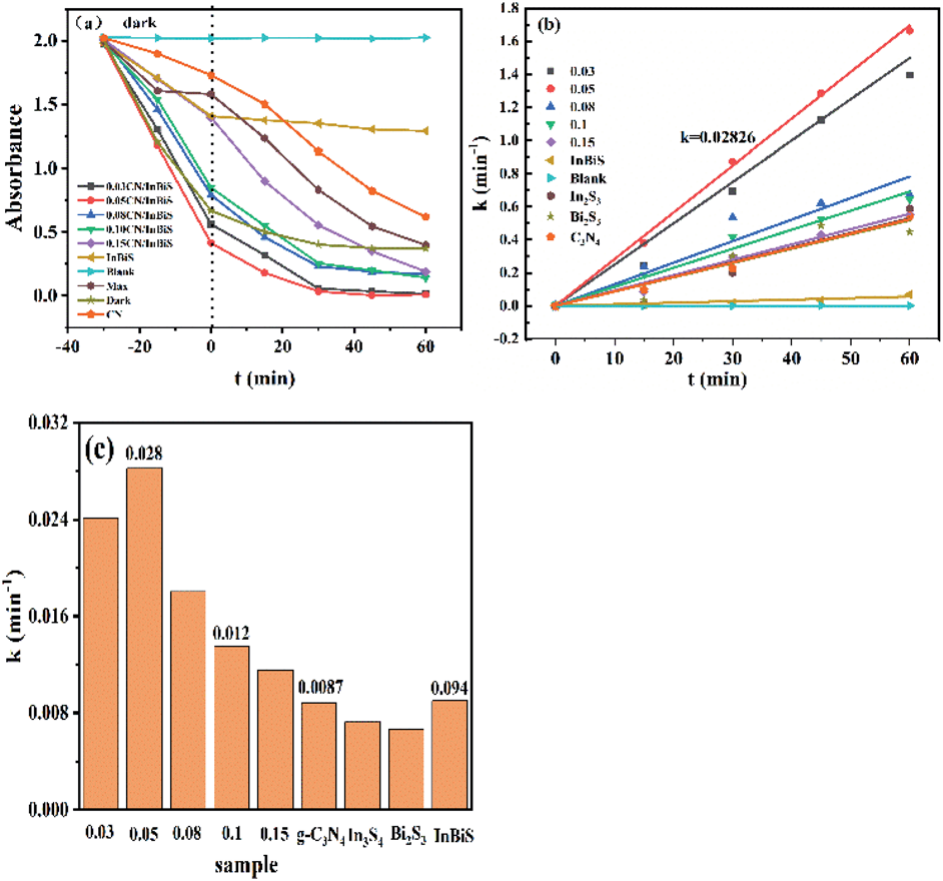
(a) Influence that the illumination time has on the photocatalytic degradation of the catalysts, (b) the pseudo-first-order dynamics, and (c) the apparent rate constant of the degradation reaction.

Fig. 7a shows the degradation RhB by the different catalysts. It can be clearly seen that the 0.05 g-C_3_N_4_ composite catalyst shows the best catalytic activity, which almost completely degrades the dye after 45 min of exposure to light. Fig. 7b shows the first-order kinetic fitting curve of the degradation, and the results obtained are consistent with the results shown in the degradation curve. Fig. 7c shows a histogram of the catalyst fitting coefficient. The fitting coefficient of the 0.05 g-C_3_N_4_/Bi_2_S_3_/In_2_S_3_ catalyst is 0.02826, which is significantly higher than the values for g-C_3_N_4_, In_2_S_3_ and Bi_2_S_3_. Compared with recently published articles, this study has advantages in degradation efficiency.^56–58^

### 3.3 Research on the mechanism of photocatalysis

**Fig. 8:**
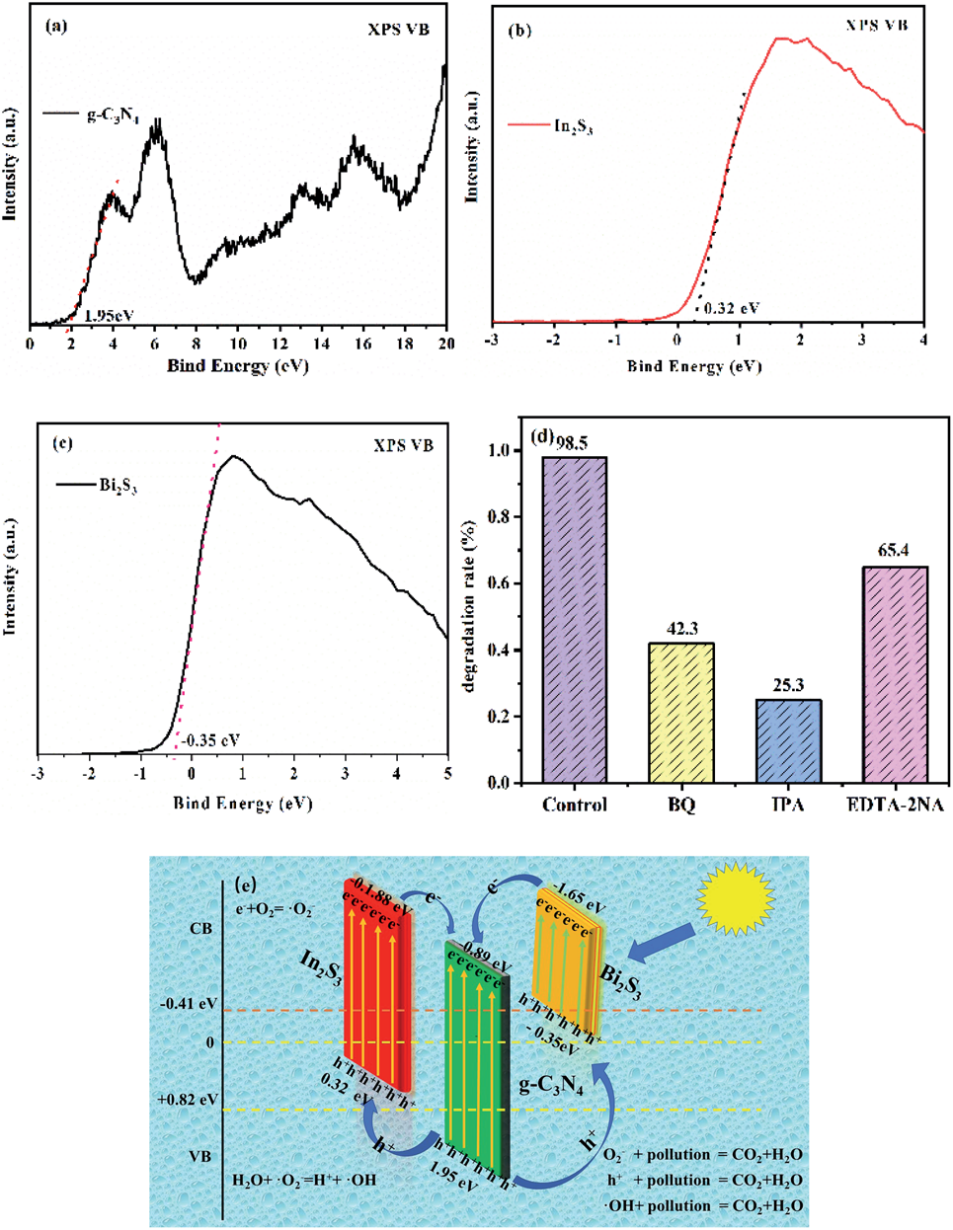
(a)–(c) show the valence band diagrams of g-C_3_N_4_, In_2_S_3_ and Bi_2_S_3_; (d) the free radical trapping experiments for the degradation of RhB over the 0.05CN/Bi_2_S_3_/In_2_S_3_ composite. (e) The proposed photocatalytic mechanism for the degradation of RhB on the surface of the 0.05CN/Bi_2_S_3_/In_2_S_3_ composite.

Fig. 8a–c show the valence band spectra of g-C_3_N_4_, In_2_S_3_ and Bi_2_S_3_ respectively (1.95, 0.32, and −0.35 eV). From the results obtained and shown in Fig. 5b, the valence (VB) and conduction bands (CB), respectively, of g-C_3_N_4_ (1.95 and −0.89 eV), In_2_S_3_ (0.32 and −1.88 eV), and Bi_2_S_3_ (−0.35 and −1.65 eV) can be calculated, which facilitate the subsequent analysis of the mechanism of the photocatalyst degradation. Fig. 8e shows the particle capture experiment performed by the catalyst, using isopropanol (IPA), 1,4-benzoquinone (BQ), and Na_2_C_2_O_4_ as the reactive species scavengers for ˙OH, ˙O_2_^−^, and h^+^, respectively.^59^ It can be seen that BQ, IPA and EDTA-2NA all have different degrees of influence on the catalytic performance of the catalyst. Among them, IPA has the greatest degree of influence, with a degradation rate of 28.3%, whereas EDTA-2Na has the lowest degree of influence and a degradation rate of 65.3%. This shows that photogenerated e^−^ have a greater impact on the ternary heterostructured nanocomposite g-C_3_N_4_/In_2_S_3_/Bi_2_S_3_ than h^+^. Fig. 8e shows the possible reaction pathway of the photogenerated carriers of the ternary heterostructured nanocomposite g-C_3_N_4_/In_2_S_3_/Bi_2_S_3_ catalyst. When the catalyst is excited by light, e^−^ transition from the VB to the CB. Due to the difference in electronegativity, the excited e^−^ will be transferred from the CBs of In_2_S_3_ and Bi_2_S_3_ to the CB of g-C_3_N_4_. At the same time, h^+^ will be transferred from the VB of g-C_3_N_4_ to the VBs of In_2_S_3_ and Bi_2_S_3_, and can also oxidise pollutants. The transfer speed of h^+^ and e^−^ is improved, and the recombination of photogenerated carriers is suppressed, which improves the photocatalytic performance of the material. It is speculated that the photocatalysis may proceed in the following way:

1 CN/Bi_2_S_3_/In_2_S_3_ + *hν* → e^−^ + h^+^

2 OH^−^ + h^+^ → ˙OH

3 H_2_O + h^+^ →H^+^ + ˙OH

4 e^−^ + O_2_ → ˙O_2_^−^

5 ˙O_2_^−^ + H^+^ → ˙HO_2_

6 2·HO_2_ → O_2_ + H_2_O_2_

7 H_2_O_2_^+^ ˙O_2_^−^ → ˙OH + OH^−^ + O_2_

Of these, (2), (4), and (7) are thought to be the main photocatalytic reactions that occur.

### 3.4 Stability testing

**Fig. 9:**
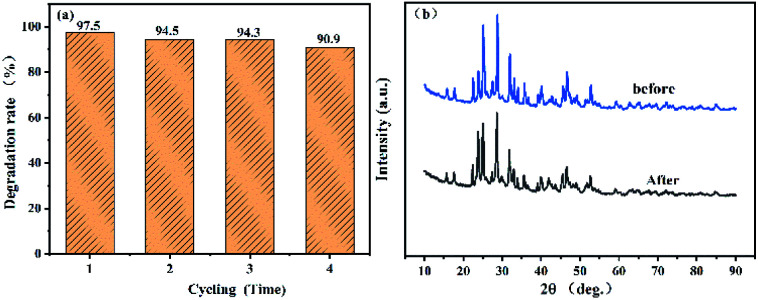
(a) Cycling runs of 0.05CN/Bi_2_S_3_/In_2_S_3_ in the degradation of RhB under visible light. (b) PXRD pattern of 0.05CN/Bi_2_S_3_/In_2_S_3_ before and after carrying out the photocatalytic experiments.

Fig. 9a shows the stability test results of the 0.05 g-C_3_N_4_/Bi_2_S_3_/In_2_S_3_ catalyst. After repeating the degradation test four times, the degradation performance of the catalyst still reaches 90.9. Therefore, it can be seen that the heterostructure of the composite catalyst not only improves its activity, but also its stability. To further assess whether the structure of the catalyst is damaged after use, PXRD measurements were carried out on the catalyst before and after its use, and the results are shown in Fig. 9b. It can be seen that there is basically no change in the PXRD peak patterns of the catalyst before and after use. Therefore, it can therefore be concluded that the catalyst structure is not destroyed after use.

## 4 Conclusions

In this paper, a one-step hydrothermal method was used to synthesise a 3D dendritic and porous spherical ternary heterostructure nanocomposite g-C_3_N_4_/In_2_S_3_/Bi_2_S_3_. The g-C_3_N_4_/In_2_S_3_/Bi_2_S_3_ heterostructure catalyst shows significantly improved degradation of RhB compared with the components from which it is made. The reason for the improved photocatalytic performance can be attributed to the increase in the specific surface area of the catalyst, meaning that it has more active sites; the formation of 3D dendritic and porous spherical materials enhances the adsorption performance of the catalyst and the composition of the heterostructure catalyst accelerates the separation of photogenerated carriers, thereby promoting the production of O_2_^−^, OH, and h^+^. The research herein describes the design of a new type of ternary heterostructure catalyst. The disadvantage here is that the decomposition process of the degradants was not able to be analysed in detail, which remains a subject for future work.

## Conflicts of interest

The authors declare that they have no competing interests.

## Supplementary Material

RA-011-D1RA00729G-s001

## References

[cit1] Wenwu Z., Beibei X., Zhiping L., Zongpeng W., Liangai H., Shijie S., Qinghua Z., Lin G. (2021). RhSe_2_: A Superior 3D Electrocatalyst with Multiple Active Facets for Hydrogen Evolution Reaction in Both Acid and Alkaline Solutions. Adv. Mater..

[cit2] Wang Z., Lin Z., Deng J., Shen S., Gu L. (2020). Elevating the d and Center of Six ordinated Octahedrons in Co_9_S_8_ through Fe ncorporated Topochemical Deintercalation. Adv. Energy Mater..

[cit3] Yang C., Zhao Z. Y., Wei H. T., Deng X. Y., Liu Q. J. (2021). DFT calculations for single-atom confinement effects of noble metals on monolayer g-C_3_N_4_ for photocatalytic applications. RSC Adv..

[cit4] Li X., Jiang H., Ma C., Zhu Z., Li X. (2021). Local surface plasma resonance effect enhanced Z-scheme ZnO/Au/g-C_3_N_4_ film photocatalyst for reduction of CO_2_ to CO. Appl. Catal., B.

[cit5] Cao M., Wang K., Tudela I., Fan X. (2021). Improve photocatalytic performance of g-C_3_N_4_ through balancing the interstitial and substitutional chlorine doping. Appl. Surf. Sci..

[cit6] Lyu L., Wang X., Sui X., Liu Z., Yuan Y., Lin C. (2018). Heavy Metal Pollution and Ecological Risk Assessment of Cultivated Land Soil in the Farming Areas of Coastal China: A Case Study of Donghai County, Jiangsu Province. Agric. Biotechnol..

[cit7] Iqbal M., Syed J. H., Breivik K., Chaudhry M. J. I., Li J., Zhang G., Malik R. N. (2017). E-Waste Driven Pollution in Pakistan: The First Evidence of Environmental and Human Exposure to Flame Retardants (FRs) in Karachi City. Environ. Sci. Technol..

[cit8] Imen G., Ines Z., Catia B., Angelo C., Ahmed G. (2018). Seasonal occurrence, source evaluation and ecological risk assessment of polycyclic aromatic hydrocarbons in industrial and agricultural effluents discharged in Wadi El Bey (Tunisia). Environ. Geochem. Health.

[cit9] Varol S., İlknur K. (2018). Effect on human health of the arsenic pollution and hydrogeochemistry of the Yazr Lake wetland (Burdur/Turkey). Environ. Sci. Pollut. Res..

[cit10] Tan H., Luo J., Orderud G. I., Zheng Y., Pan J. J., Studies E. (2015). The Pollution Caused by Protection: The Unintended Consequences of the Local Governance of the Urban Drinking Water Source Protection in Tianjin, China. Chinese Journal of Urban and Environmental Studies.

[cit11] Kahn M. E., Li P., Zhao D. (2015). Water Pollution Progress at Borders: The Role of Changes in China's Political Promotion Incentives. American Economic Journal.

[cit12] Wu F., Feng C., Zhang R., Li Y., Du D. (2012). Derivation of water quality criteria for representative water-body pollutants in China. Sci. China: Earth Sci..

[cit13] Tan W., Wang Y., Sun S., Wu F. (2011). Zeolite for removal of organic micro-pollutants in water. Miner. Process..

[cit14] Fei W., Ming H., Jinsheng L., Peizhang G., Maomao Z., Baizeng F., Hui Z., Zengyao S. (2019). A facile fabrication of sepiolite mineral nanofibers with excellent adsorption performance for Cd^2+^ ions. RSC Adv..

[cit15] Hassan M., Olvera-Vargas H., Zhu X., Zhang B., He Y. (2019). Microbial electro-fenton: an emerging and energy-efficient platform for environmental remediation. J. Power Sources.

[cit16] Guangfu L., Chunxue L., Xinzheng L., Baizeng F. (2021). Emerging polymeric carbon nitride Z-scheme systems for photocatalysis. Cell Rep. Phys. Sci..

[cit17] Chaoran J., Ki Yip L., Christopher M. A. P., Mustafa K. B., Chi Ching L., Qiushi R., Savio J. A. M., Adam F. L., Junwang T. (2015). Size-controlled TiO_2_ nanoparticles on porous hosts for enhanced photocatalytic hydrogen production. Appl. Catal., A.

[cit18] Ning A., Yuwei M., Juming L., Huiyan M., Jucai Y., Qiancheng Z. (2018). Enhanced visible-light photocatalytic oxidation capability of carbon-doped TiO_2_*via* coupling with fly ash. Chin. J. Catal..

[cit19] Zhenwei T., Dong Y., Tianxiong X., Yao T., Zhongyi J. (2015). Biomimetic fabrication of g-C_3_N_4_/TiO_2_ nanosheets with enhanced photocatalytic activity toward organic pollutant degradation. Chem. Eng. J..

[cit20] Xin W., Yuren X., Benqing Z., Youming Z., Jiatao W., Rui H., Liwei L., Jun S., Junle Q. (2019). Enhanced photocatalytic performance of Ag/TiO_2_ nanohybrid sensitized by black phosphorus nanosheets in visible and near-infrared light. J. Colloid Interface Sci..

[cit21] Fenfen Z., Yuefei R., Junmin W., Zhiwen H., Zhiqin P., Bing W. (2018). MoS_2_ quantum dots@TiO_2_ nanotube composites with enhanced photoexcited charge separation and high-efficiency visible-light driven photocatalysis. Nanotechnology.

[cit22] Ruonan G., Guixian Z., Yingjie G., Bo L., Jianfeng G., Xiuwen C. (2019). Synthesis of 3D Bi_2_S_3_/TiO_2_ NTAs photocatalytic system and its high visible light driven photocatalytic performance for organic compound degradation. Sep. Purif. Technol..

[cit23] Pham Van T., Tran Thi P., Vu Thi T., Sang Xuan N., Tran Ngoc K. (2020). In situ hydrothermal fabrication and photocatalytic behavior of ZnO/reduced graphene oxide nanocomposites with varying graphene oxide concentrations. Mater. Sci. Semicond. Process..

[cit24] Zhichao S., Zhiquan Y., Yingya L., Chuan S., Mingshan Z., Anjie W. (2019). Construction of 2D/2D BiVO_4_/g-C_3_N_4_ nanosheet heterostructures with improved photocatalytic activity. J. Colloid Interface Sci..

[cit25] Liao G., Gong Y., Zhang L., Gao H., Yang G. J., Fang B. (2019). Semiconductor polymeric graphitic carbon nitride photocatalysts: the “holy grail” for the photocatalytic hydrogen evolution reaction under visible light. Energy Environ. Sci..

[cit26] Liao G., He F., Li Q. (2020). *et al.*, Emerging graphitic carbon nitride-based materials for biomedical applications. Prog. Mater. Sci..

[cit27] Fei C., Wenjing Y., Wenbo C., Feiyan W., Baoqing D., Xuefeng H. (2018). The construction and enhanced photocatalytic performance of binary composite S/g-C_3_N_4_. Mater. Sci. Semicond. Process..

[cit28] Natkritta B., Natda W., Sukon P., David W., Peter S., Andrew N., Jun C., Burapat I. (2014). Enhanced visible-light photocatalytic activity of g-C_3_N_4_/TiO_2_ films. J. Colloid Interface Sci..

[cit29] Yaping W., Yike L., Jingli Z., Jianshe W., Zhongjun L. (2018). g-C_3_N_4_/B doped g-C_3_N_4_ quantum dots heterojunction photocatalysts for hydrogen evolution under visible light. Int. J. Hydrogen Energy.

[cit30] Al-Hajji L., Adel A. I., Atitar M. F., Abdelfattah I., Ahmed Mohamed E.-T. (2018). Construction of mesoporous g-C_3_N_4_/TiO_2_ nanocrystals with enhanced photonic efficiency. Ceram. Int..

[cit31] Wu X., Cao L., Song J., Si Y., Ding B. (2020). Thorn-like flexible Ag_2_C_2_O_4_/TiO_2_ nanofibers as hierarchical heterojunction photocatalysts for efficient visible-light-driven bacteria-killing. J. Colloid Interface Sci..

[cit32] Liu X., Gu S., Zhang X., Li X., Li W. (2021). The production discipline and mechanism of hydroxyl radical by investigating the Ln_2_O_3_-Bi_2_MoO_6_ heterojunction photocatalysts. J. Alloys Compd..

[cit33] Katsumata K. I., Motoyoshi R., Matsushita N., Okada K. (2013). Preparation of graphitic carbon nitride (g-C_3_N_4_)/WO_3_ composites and enhanced visible-light-driven photodegradation of acetaldehyde gas. J. Hazard. Mater..

[cit34] Junqing Y., Huan W., Hong C., Yunxia Z., Fuxiang Z., Shengzhong Frank L. (2016). Fabrication of TiO_2_/C_3_N_4_ heterostructure for enhanced photocatalytic Z-scheme overall water splitting. Appl. Catal., B.

[cit35] Wang J., Zhang W. D. (2012). Modification of TiO_2_ nanorod arrays by graphite-like C_3_N_4_ with high visible light photoelectrochemical activity. Electrochim. Acta.

[cit36] Yang G., Jinhai L., Zhanqi G., Xin Z., Ying L., Zhongbo W., Wei Z., Cheng S. (2016). A simple and effective method for fabricating novel p–n heterojunction photocatalyst g-C_3_N_4_/Bi_4_Ti_3_O_12_ and its photocatalytic performances. Appl. Catal., B.

[cit37] Chunmei L., Siyu Y., Hongjun D., Chunbo L., Haijun W., Huinan C., Gang C. (2018). Z-scheme mesoporous photocatalyst constructed by modification of Sn_3_O_4_ nanoclusters on g-C_3_N_4_ nanosheets with improved photocatalytic performance and mechanism insight. Appl. Catal., B.

[cit38] Auttaphon C., Watcharapong P., Tawanwit L., Titipun T., Somchai T., Sila K., Sulawan K. (2019). Enhanced photocatalytic degradation of methylene blue by a direct Z-scheme Bi_2_S_3_/ZnIn_2_S_4_ photocatalyst. Mater. Res. Bull..

[cit39] Geioushy R. A., El-Sheikh S. M., Ahmed B. A., Bahaa Ahmed S., Farida M. E.-D. (2020). One-pot fabrication of BiPO_4_/Bi_2_S_3_ hybrid structures for visible-light driven reduction of hazardous Cr(vi). J. Hazard. Mater..

[cit40] Yagna P. B., Dibyananda M., Krishnendu D., Braja G. M. (2019). Visible Light Assisted Photocatalytic Degradation of Phenolic Compounds Using Bi_2_S_3_/Bi_2_W_2_O_9_ Heterostructure Materials as Photocatalyst. Chemistryselect.

[cit41] Xiaoqiang A., Jimmy C. Y., Feng W., Chuanhao L., Yecheng L. (2013). One-pot synthesis of In_2_S_3_ nanosheets/graphene composites with enhanced visible-light photocatalytic activity. Appl. Catal., B.

[cit42] Zhong W., Shen S., Feng S., Lin Z., Wang Z., Fang B. (2018). Facile fabrication of alveolate Cu_2−*x*_ Se microsheets as a new visible-light photocatalyst for discoloration of rhodamine B. CrystEngComm.

[cit43] Guangfu L., Jiasheng F., Qing L., Sihan L., Zushun X., Baizeng F. (2019). Ag-based Nanocomposites: Synthesis and Applications in Catalysis. Nanoscale.

[cit44] Samsudin M. F. R., Dumas A., Bashiri R., Mohammed N. M., Sufian S. J. (2020). Development of the g-C_3_N_4_/BiVO_4_ Microflower Photocatalyst for Photocatalytic Degradation of Amoxicillin and Hydrogen Production. Malaysian Journal of Microscopy.

[cit45] Jinshan H., Pengfei Z., Jifang C., Weijia A., Li L., Yinghua L., Qingbin Y., Hongjun Y., Wenquan C. (2020). High-efficiency removal of phenol and coking wastewater *via* Photocatalysis-Fenton synergy over a Fe-g-C_3_N_4_ graphene hydrogel 3D structure. J. Ind. Eng. Chem..

[cit46] Jing F., Tingting C., Shenna L., Qihang Z., Yueming R., Yanzhuo L., Zhuangjun F. (2016). Improvement of g-C_3_N_4_ photocatalytic properties using the Hummers method. J. Colloid Interface Sci..

[cit47] Xingzhong Y., Longbo J., Jie L., Yang P., Jin Z., Hou W., Lijian L., Zhibin W., Renpeng G., Guangming Z. (2019). In situ synthesis of 3D microsphere-like In_2_S_3_/InVO_4_ heterojunction with efficient photocatalytic activity for tetracycline degradation under visible light irradiation. Chem. Eng. J..

[cit48] Qiang H., Ci'an X., Yongming H., Daimei C., Yiwen L., Wei W., Bing-Jie N. (2020). Accelerated separation of photogenerated charge carriers and enhanced photocatalytic performance of g-C_3_N_4_ by Bi_2_S_3_ nanoparticles. Chin. J. Catal..

[cit49] Xue Song Z., Jian-Yang H., Hong J. (2014). Facile modification of a graphitic carbon nitride catalyst to improve its photoreactivity under visible light irradiation. Chem. Eng. J..

[cit50] Min Quan Y., Bo W., Yi-Jun X. (2013). Synthesis of In_2_S_3_–CNT nanocomposites for selective reduction under visible light. J. Mater. Chem. A.

[cit51] Jinze L., Yue M., Zhefei Y., Mingjun Z., Huiqin W., Changchang M., Dongdong W., Pengwei H., Yongsheng Y. (2017). Fast electron transfer and enhanced visible light photocatalytic activity using multi-dimensional components of carbon quantum dots@3D daisy-like In_2_S_3_/single-wall carbon nanotubes. Appl. Catal., B.

[cit52] Yanyan W., Xin D., Ping Z., Qi W., Kang Z., Lin C., Jianjun D., Xingyou T., Xian Z. (2019). Convenient and Recyclable TiO_2_/g-C_3_N_4_ Photocatalytic Coating: Layer-by-layer Self-assembly Construction on Cotton Fabrics Leading to Improved Catalytic Activity under Visible Light. Ind. Eng. Chem. Res..

[cit53] Guanglan D., Zhiliang Z., Qinghui H., Hua Z., Jianyao Z., Yanling Q., Daqiang Y., Jianfu Z. (2018). Targeted modulation of g-C_3_N_4_ photocatalytic performance for pharmaceutical pollutants in water using ZnFe-LDH derived mixed metal oxides: Structure-activity and mechanism. Sci. Total Environ..

[cit54] Guiming P., Lidan X., Jesús B., Michael V., Menny S. (2018). Frontispiz: A General Synthesis of Porous Carbon Nitride Films with Tunable Surface Area and Photophysical Properties. Angew. Chem..

[cit55] Huang Y., Fan W., Long B., Li H., Zhao F., Liu Z., Tong Y., Ji H. (2016). Visible light Bi_2_S_3_/Bi_2_O_3_/Bi_2_O_2_CO_3_ photocatalyst for effective degradation of organic pollutions. Appl. Catal., B.

[cit56] Chao L., Lili W., Xiaogang L., Lu Z., Xuefang L., Jinsheng S. (2020). An efficient inverse opal (IO)-TiO_2_-MoO_3−*x*_ for photocatalytic H_2_ evolution and RhB degradation – The synergy effect of IO structure and plasmonic MoO_3−*x*_. Appl. Surf. Sci..

[cit57] Xiaoxiao L., Qiang L., Shihao L., Rui L., Hong L., Min Z., Chaopeng C., Guangping Z., San C., Changhao L. (2020). Fabrication of a novel BiOI/KTaO_3_ p–n heterostructure with enhanced photocatalytic performance under visible-light irradiation. RSC Adv..

[cit58] Zhangfeng S., Qiulin Z., Chaochuang Y., Shifei K., Hongyan J., Xing L., Xi L., Yangang W., Lifeng C. (2019). Facile synthesis of 3D flower-like mesoporous Ce–ZnO at room temperature for the sunlight-driven photocatalytic degradations of RhB and phenol. J. Colloid Interface Sci..

[cit59] Tingjiang Y., Jun T., Wenfei G., Zheng Q., Wenjuan L., Jinmao Y., Baibiao H. (2017). Ultra-low loading of Ag_3_PO_4_ on hierarchical In_2_S_3_ microspheres to improve the photocatalytic performance: the cocatalytic effect of Ag and Ag_3_PO_4_. Appl. Catal., B.

