# Supplementary material for: One-step hydrothermal synthesis of a ternary heterojunction g-C3N4/Bi2S3/In2S3 photocatalyst and its enhanced photocatalytic performance
Source: RSC Adv. 2021 Mar 5;11(17):9788–96. doi: 10.1039/d1ra00729g (PMC8695387; doi:10.1039/d1ra00729g)
Supplement: RA-011-D1RA00729G-s001 [file RA-011-D1RA00729G-s001.pdf]

Electronic Supplementary Material (ESI) for RSC Advances.
This journal is © The Royal Society of Chemistry 2021

Additional information

Due to limited laboratory equipment and the impact of the epidemic, the TOC curve cannot be measured

We have adopted another method that can be implemented.

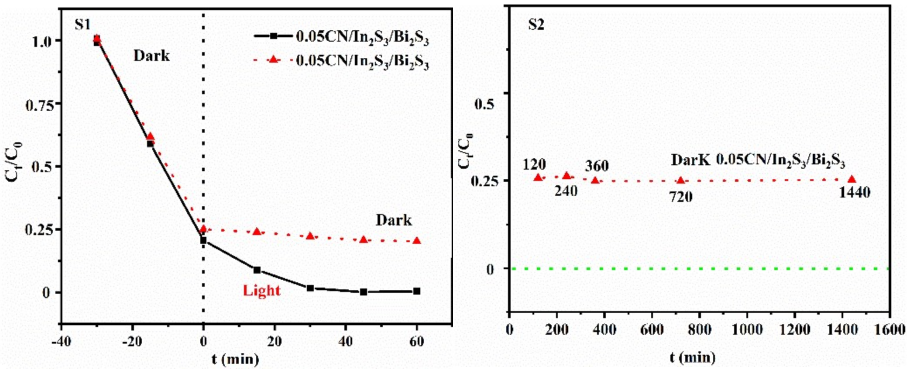

As shown in Figure S_1, S_2, the same catalyst was used for comparison under dark and light conditions. The initial concentration ratio of the solution under the dark condition for a long time (12h) is kept at 0.25. However, after 15 minutes of light conditions, the absorbance of the solution was almost zero. It can be inferred that the content of organic matter in the solution is decreasing under light conditions. (Because the absorbance of azo dyes is related to the concentration at a specific wavelength)
